# Dynamics of plastic debris and its density change between river compartments in the Tuul River system, Mongolia

**DOI:** 10.1007/s11356-024-35584-w

**Published:** 2024-11-26

**Authors:** Dolgormaa Munkhbat, Batdulam Battulga, Bolormaa Oyuntsetseg, Masayuki Kawahigashi

**Affiliations:** 1 Department of Geography, Tokyo Metropolitan University, 1-1 Minami-Osawa, Hachioji, Tokyo, 192-0397 Japan; 2 Nuclear Science and Engineering Center, Japan Atomic Energy Agency, Tokai, Ibaraki, 319-1195 Japan; 3 Department of Chemistry, National University of Mongolia, Ikh Surguuliin Gudamj-1, Ulaanbaatar, 14201 Mongolia

**Keywords:** Microplastic, Macroplastic, Plastic density change, Carbonyl index, Photodegradation, River system, Plastic aging

## Abstract

Plastic pollution in river environments has become an emerging global concern. However, the migration of plastic and changes in its properties between river compartments are less understood. This study demonstrates the plastic debris aging and the dynamics between floodplain, surface water, and sediment compartments of the Tuul River, Mongolia. Plastic occurrence is evaluated in terms of their abundance, size, shape, polymer type, and photodegradation in each compartment. Photodegradation stages were calculated using the carbonyl index (CI). Plastic abundance was 5.46 ± 3.53 items m^−2^ in the floodplain, 155 ± 100.7 items m^−3^ in the surface water, and 128.4 ± 76.3 items kg^−1^ in the sediment. Microplastics dominated in the size category in all compartments, while macro- and megaplastics were found only in the floodplain. Polyethylene and polypropylene dominated the surface water and sediment, while polystyrene was the predominant plastic in the floodplain. A positive correlation was found between the distributed polymer types in the surface water and sediment compartments. The similar composition in size and polymer type suggests vertical plastic migration from water to sediment. Although CI values showed that the plastic aging was significantly different between water and sediment (water, 0.61 ± 0.26, and sediment, 0.90 ± 0.68), the dominance of low-density plastics with high CI in the sediment suggests that the aged plastic density changed during the vertical transport in the river system.

**Supplementary information:**

The online version contains supplementary material available at 10.1007/s11356-024-35584-w.

## Introduction

Plastic debris in the environment is a serious threat to our future because it contaminates the air, water, and soil. The global annual production of plastic resins and chemical fibers was two million tons in the 1950s (Geyer et al. 2017). By 2022, the production of all types of plastics increased to 400.3 million tons (PlasticEurope, 2023). This rapid growth of the plastic industry is related to economical production processes and extraordinarily convenient properties, leading to overuse, intentional and unintentional waste disposal, and fatal environmental pollution coupled with ecosystem disturbance on a global scale.

UNESCO reported that 80% of marine pollution consisted of plastic waste, which may have ended up in the ocean from land (UNESCO 2023). Lebreton et al. (2017) have estimated that 1.15–2.41 million tons of plastic debris enter the ocean from rivers. Rivers play key roles in controlling plastic debris from both qualitative and quantitative perspectives. Tracking the plastic transport process in the river system is a significant challenge in simulating the dynamics of released plastic debris in the aquatic environment.

The plastic distribution in river compartments is evaluated by several different sampling methods. Plastic on a riverbank or floodplain area is visually collected, and the quantity is expressed as plastic items per area (Bruge et al. 2018; Battulga et al. 2020; Bernardini et al. 2020). Floating plastics in surface river water are often evaluated using trapping nets, such as manta trawl and plankton nets, direct grab sampling (Barrows et al. 2017), and visual observation methods (van Emmerik et al. 2019; Gallitelli and Scalici 2022). Sedimentary plastics are collected using a shovel or grab samplers (Razeghi et al. 2021); thereafter, density separation and spectroscopic analyses are conducted.

During transport in a river, plastic debris is separated according to its properties, such as size, polymer type, hydrophobicity, and density (Zhang et al. 2020; Ren et al. 2021). These properties are the driving force of its flow and retention rate in the river system. Micro- (Li et al. 2023) and macroplastic distribution (Al-Zawaidah et al. 2021) (considered macro > 0.5 cm) in global river systems has been reported, although there are some gaps in the literature. First, most plastic studies have focused only on the microplastic size category in freshwater systems, and few studies report the distribution of other size scale categories. Second, the interrelationship between the river compartments of surface water and sediment has not been discussed, and it is necessary to understand microplastic formation processes and the behavior of microplastic in-stream processes. This is because the simultaneous sampling of plastic debris in river surface water and sediment is necessary in a wide range of rivers. Although the composition of microplastics in river waters and sediments from the viewpoint of the abundance, size, shape, and polymer composition has been reported in most studies (Blair et al. 2019; Xiong et al. 2019; Li et al. 2023; Akdogan et al. 2023), changes in the degradation stages of plastics between river compartments have not yet been explained. Understanding the dynamics of plastics ranging from micro to macrosizes in floodplains, river water, and sediments as an entire river system can fill a significant gap in the existing literature (Strokal et al. 2023).

Common plastics found in river bottom sediments are polyethylene and polypropylene (Curren et al. 2021; Bai et al. 2022), which have lower densities than water, indicating that there must be a changing process of the apparent plastic density (Zhang et al. 2020) in-stream processes. These plastic interrelationships between water and sediment compartments may be related to the various degradation processes of plastic debris.

When released plastic debris reaches the aquatic systems, it undergoes various reactions, such as photooxidation, microbial colonization, biofouling, and thermal and mechanical degradation (Andrady and Koongolla 2022; Devi et al. 2023). Among these reactions, photooxidation has a stronger impact on plastic degradation because it strongly damages chemical bonding by solar ultraviolet radiation (Andrady and Koongolla 2022). The structural properties of plastic debris lead to further reactions with other organic and mineral substances on the surface. The photodegradation process is often referred to as a photoaging (Guo et al. 2023; Syranidou et al. 2023; Wang et al. 2023) and it can be evaluated from their infrared (IR) spectra using the carbonyl index (CI). The CI calculation is based on the formation of carbonyl groups in the polymer chain structure. Differences in the CI values enable the tracking of degradation processes during transgression within river compartments.

We study the dynamics and distribution of plastic debris in three compartments of river systems: floodplain, surface water, and sediment. The size, shape, material composition, and photodegradation in the river compartments are investigated.

## Materials and methods

### Regional background

Mongolia is a landlocked country, and its population is concentrated in the capital city, Ulaanbaatar. The city center has been developed along the Tuul River, which runs from the east to the west border of the city. The increase in population in Ulaanbaatar during the last three decades has promoted the development of the suburban hilly areas surrounding the city for residential, industrial, and agricultural areas. Additionally, landfills have been created on a large scale, resulting in urban waste, including plastic waste, being collected at landfills. Plastic debris released from dumping sites by wind and leaked during the transport process gathered at lower elevation and finally reached the Tuul River. Mongolia has four seasons and endures a harsh continental climate with high-temperature fluctuations. The minimum and maximum temperatures in Ulaanbaatar city ranged from − 39.8 to + 38.3 °C between 1991 and 2020, according to the Mongolian Meteorological Agency (NAMEM) archives (NAMEM et al., 2023). The major land use of Ulaanbaatar city is divided into residential areas (45.4%), water protection zones (20.1%), greenery areas (15.6%), industrial areas (7.2%), and others (11.7%).

### Study area

**Fig. 1:**
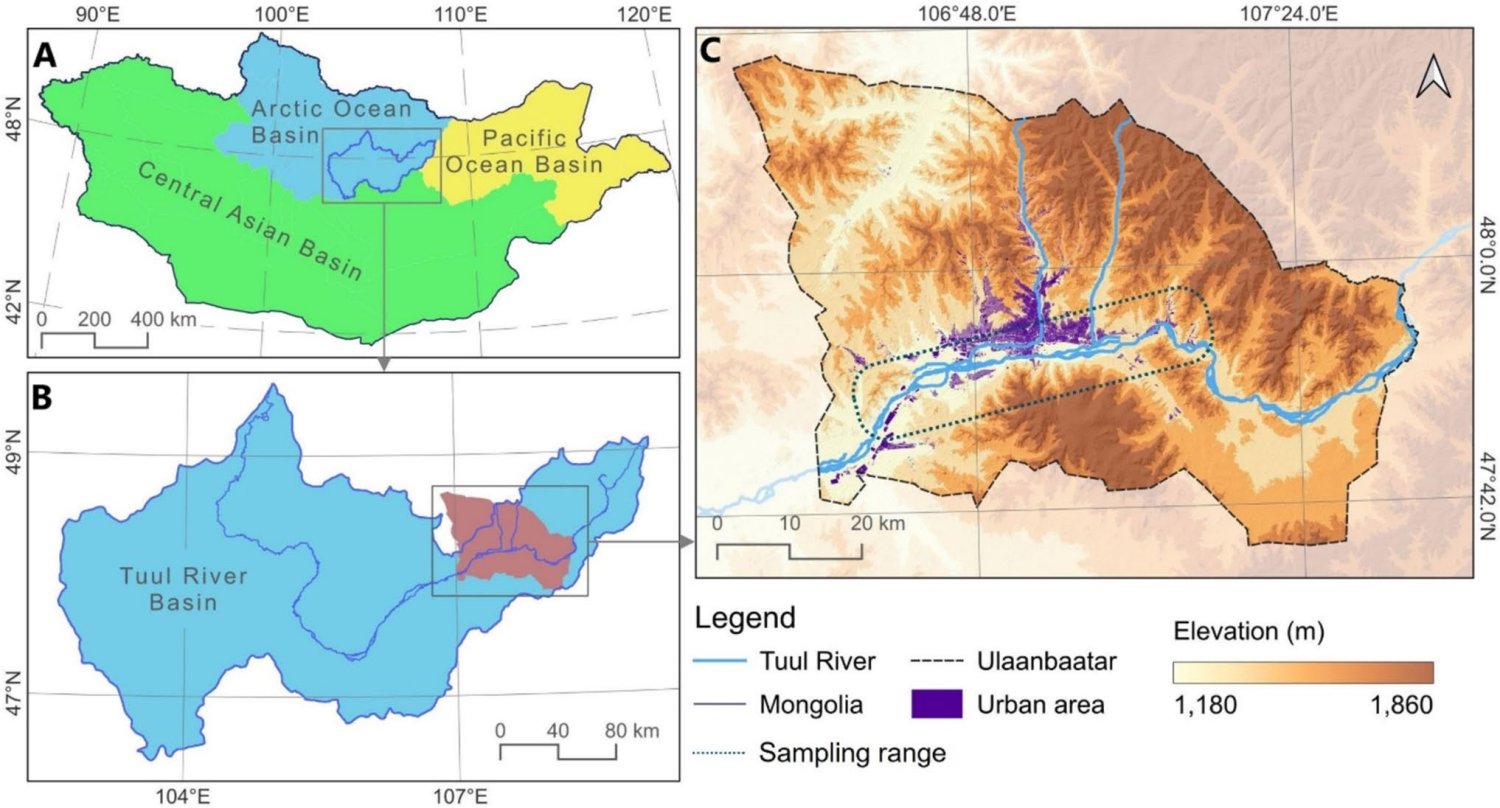
Study area map. **A** Mongolian territory. **B** Tuul River Basin. **C** Ulaanbaatar city area

The field survey and sample collection were conducted along the Tuul River and its tributaries flowing in the center of Ulaanbaatar (Fig. 1). The river was relatively shallow (0.3–3.5 m) and changed seasonally (average of 0.6 m from March to April, 1.3 m from May to Nov, and frozen from Dec to Feb) (Batsaikhan et al. 2018). The river bottom is predominantly composed of gravel beds. However, spring floods sometimes lead to river shore degradation, resulting in fine particle sediment accumulation along the river shore. Fine sediments in the littoral zone along the river shore collect plastic debris and retain them in the bottom sediment. The river environment is surrounded by urban areas, and the floodplain is covered with sparse vegetation.

### Sample collection

Plastic debris samples were collected from nine sampling sites in the city of Ulaanbaatar along the Tuul River system (Fig. 1C), including the floodplain, river surface water, and sediment compartments. These sites were selected to understand the general distribution of plastic debris in the three compartments of the urban river system. The sampling sites are considered replicates within each compartment to address plastic debris contamination and accumulation in the urban river. The range of sampling area is located in the central area of Ulaanbaatar (Fig. 1C).

The plastic debris in the floodplain was visually collected from triplicate quadrates with an area of 50 m^2^. The collected plastic debris was counted according to size fractions (micro, below 0.5 cm; meso, 0.5–2 cm; macro, 2–10 cm; and mega, above 10 cm) and separated by the types of shape (films, fragments, fibers, and foams).

Plankton nets were used to collect the flowing plastic debris at sampling sites in the river. The nets sufficiently sunk into the water body during the surface water sampling and kept it for a fixed time frame on sampling sites, coupled with flow rate measurements using a flow meter (KENEK corp. Tokyo JAPAN). The mesh size of the net was 300 µm. Suspended solids collected in a vessel attached to the bottom of the plankton net were immediately transferred to the sample container and stored in a refrigerator until sample analysis.

River sediments were collected from the littoral zone along the river shore using a stainless-steel shovel at sampling sites and directly stored in a sterilized tube (B.D Falcon, Switzerland). The sediment sampling depth was 15 cm from the surface sediment.

### Sample analyses

The floodplain plastic samples were digested with hydrogen peroxide solution (30%) to decompose the attached organic matter and remove surface contaminants such as dirt and mud. A micro-Fourier-transform IR (FTIR) instrument (AIM-8800, Shimadzu) was used to identify the digested plastic debris in the reflectance mode, focusing on a fixed aperture size (100 µm × 100 µm). The scanning range was between 400 and 4000 cm^−1^. The scanning time was 100 s per spectroscopic operation. Identifiable spectra were selected from several focusing areas in one plastic debris sample on the sample stage.

The suspended solids in the plankton net vessel were collected on a glass fiber filter (GF/F), followed by rinsing with deionized water and drying in an oven. Thereafter, the dried samples were weighed in a tall beaker and digested using a hydrogen peroxide solution (30%). Afterward, the sodium polytungstate solution (1.4 g/cm^3^) was added to the digested sample and shaken for 2 h at 500 rpm in a laboratory shaker. Subsequently, the solution was converted into a sterilized tube for centrifugation at 2000 rpm for 10 min. The supernatant solution was filtered on a 0.7 µm GF/F. During filtration, the trapped plastic materials were rinsed with distilled water several times. The GF/F was dried overnight in a dry oven at 40 °C.

Sediment samples were freeze-dried overnight and weighed in the same manner as the plastic debris in the plankton net samples. The experiments were conducted in triplicate for all analyses.

The plastic items on the dried filters were counted using a digital microscope to calculate the number of plastic debris in the samples. Thereafter, the plastic debris on the filter was identified by micro-FTIR focusing on a fixed area in the same manner as the plastic debris collected from the floodplain. The aperture size of the Micro-FTIR was 100 µm × 100 µm for most samples and 25 µm × 100 µm for some fiber samples. The carbonyl index is calculated using the ratio of absorbance intensity (Abs) between carbonyl (1715–1730 cm^−1^) and methyl (1450–1465 cm^−1^) groups as following Eq. 1.

$$\text{CI } = \frac{{\text{Abs }}_{\left({1715}-{1730}\right)}}{{\text{Abs }}_{\left({1450}-{1465}\right)}}$$

### Quality assurance and quality control

To minimize laboratory contamination, nonplastic materials such as laboratory glassware and stainless steel tweezers were used for the sample collection and laboratory experiments, except for plankton nets and falcon tubes. Cotton laboratory coats were worn during all laboratory analyses. Digestion was performed in a closed tube with a watch glass to avoid airborne contamination of the sample.

### Data analysis

One-way analysis of variance (ANOVA) was used to calculate the statistical significance between different groups of samples. Pearson’s correlation analysis was used to analyze the relationship between different polymer types of plastics in each compartment. *p*-values of < 0.05 were statistically significant. Floodplain plastic debris was expressed as plastic items per square meter area, and sediment plastic samples were expressed as items per dry weight of sediment. River discharge was calculated using river width, depth, and flow rate. Flowing plastics were expressed as items per cubic meter of river water volume. Mean abundance of plastic was expressed as mean ± STD (standard deviation).

## Result

### Plastic debris abundance

The mean value was 5.46 ± 3.53 items m^−2^ on the floodplain. The plastic distribution in riverbanks reported in some studies was 120,632 items in 8411 m^2^ (≈14.34 items m^−2^) in the Adour River, Southwest France (Bruge et al. 2018), and 27.70 items m^−2^ in the Thames River, South England (Bernardini et al. 2020). Although these results are higher than ours, they may vary depending on the size of the river and population density.

Plastics in surface water have an average value of 155 ± 100.7 items m^−3^. During the sampling, river discharges ranged between 6.9 and 29.9 m^3^ s^−1^. No statistically significant correlation was observed between the river discharge rate and the number of plastic items in surface water. We compared our results with those of other river surface water studies conducted using net sampling (Table 1). Some previous studies showed lower average abundance, such as in 29 Japanese rivers (Kataoka et al. 2019); urban Rivers, Chicago (McCormick et al. 2014); and Rhône River, France (Constant et al. 2020) as shown in Table 1. The results for the Saigon River (fragments, 10–223 items m^−3^), Vietnam (Lahens et al. 2018), and Taichung Rivers, Taiwan (Kunz et al. 2023) were closer to our results. Furthermore, some rivers showed higher amounts of plastic distribution, such as the Yangtze River, China (Zhao et al. 2014), and the Saigon River, (fibers, 172,000–519,000 item m^−3^) Vietnam (Lahens et al. 2018).

**Table 1:** The reported plastic concentration, items/m^3^ in river surface water

Study areas	Dominant plastic types	Plastic concentration, mean ± SD	Net mesh size (μm)	References
29 Rivers, Japan	PE, PP, and PS	1.6 ± 2.3	335	Kataoka et al. (2019)
Urban rivers, Chicago, USA	n.r	1.94 ± 0.81, upstream 17.9 ± 11.0, downstream	333	McCormick et al. (2014)
Rhône river	PES and PE	18.8 ± 28.1	333	Constant et al. (2020)
Saigon River, Vietnam	PE, and PP	10–223^†^, fragment 172,000–519,000^†^, fibers	300	Lahens et al. (2018)
Taichung rivers, Taiwan	PE, and PP	230, Urban area	300	Kunz et al. (2023)
Yangtze River, China	PE, and PP	800.0 ± 300.0 - 3088.9 ± 330.6^†^	48	He et al. (2021)

^†^Min–max

The sediment samples demonstrated an average concentration of 128.4 ± 76.3 items kg^−1^. Based on published global data (Table 2), we classified the plastic concentration in river sediment into three categories: low (up to 100 items kg^−1^), moderate (100–1000 items kg^−1^), and high (above 1000 items kg^−1^). Some studies reported low plastic accumulation, such as the Ciwalengke River, Indonesia (Alam et al. 2019), and Po River, Italy (Piehl et al. 2019). The high concentration of plastic in the sediment reached up to four orders of magnitude of items in 1 kg of sediment (Hurley et al. 2018; Frei et al. 2019; Singh et al. 2022). Despite these low and high values, plastic density in sediment generally fluctuates within two orders of magnitude on a 1-kg weight basis. Our result was in the moderate category.

**Table 2:** The reported plastic concentration, items/kg in other river sediments

Study areas	Predominant plastic types	Plastic concentration, min–max	References
Ciwalengke River, Indonesia	Polymer mixture and PES	30.3 ± 15.9^†^	Alam et al. (2019)
Po River, Italy	PE and PS	2.92–23.30	Piehl et al. (2019)
Romanian Danube River	PET, PP, and PS	159.2 ± 138.4^†^	Pojar et al. (2021)
Antua River, Portugal	PE and PP	18–629	Rodrigues et al. (2018)
The River Kelvin, Glasgow, UK	PE, PP, and PS	161–432	Blair et al. (2019)
Vistula River, Poland	PS, PP, and PE	190–580	Sekudewicz et al. (2021)
River Thames tributaries, UK	Organic dye, PET, and PP	185–660	Horton et al. (2017)
Ergene River, Turkey	PS and PE	97.90–277.76	Akdogan et al. (2023)
Qinhuai River, China	PP, PS, and PE	1115–6380	Yan et al. (2021)
Nakdong River, Korea	PE and PP	1971 ± 62^†^	Eo et al. (2019)
Pearl River, China	PE and PP	1669^‡^	Lin et al. (2018)

^†^Mean ± STD

^‡^Mean

### Plastic composition changes in size, shape, and polymer types

**Fig. 2:**
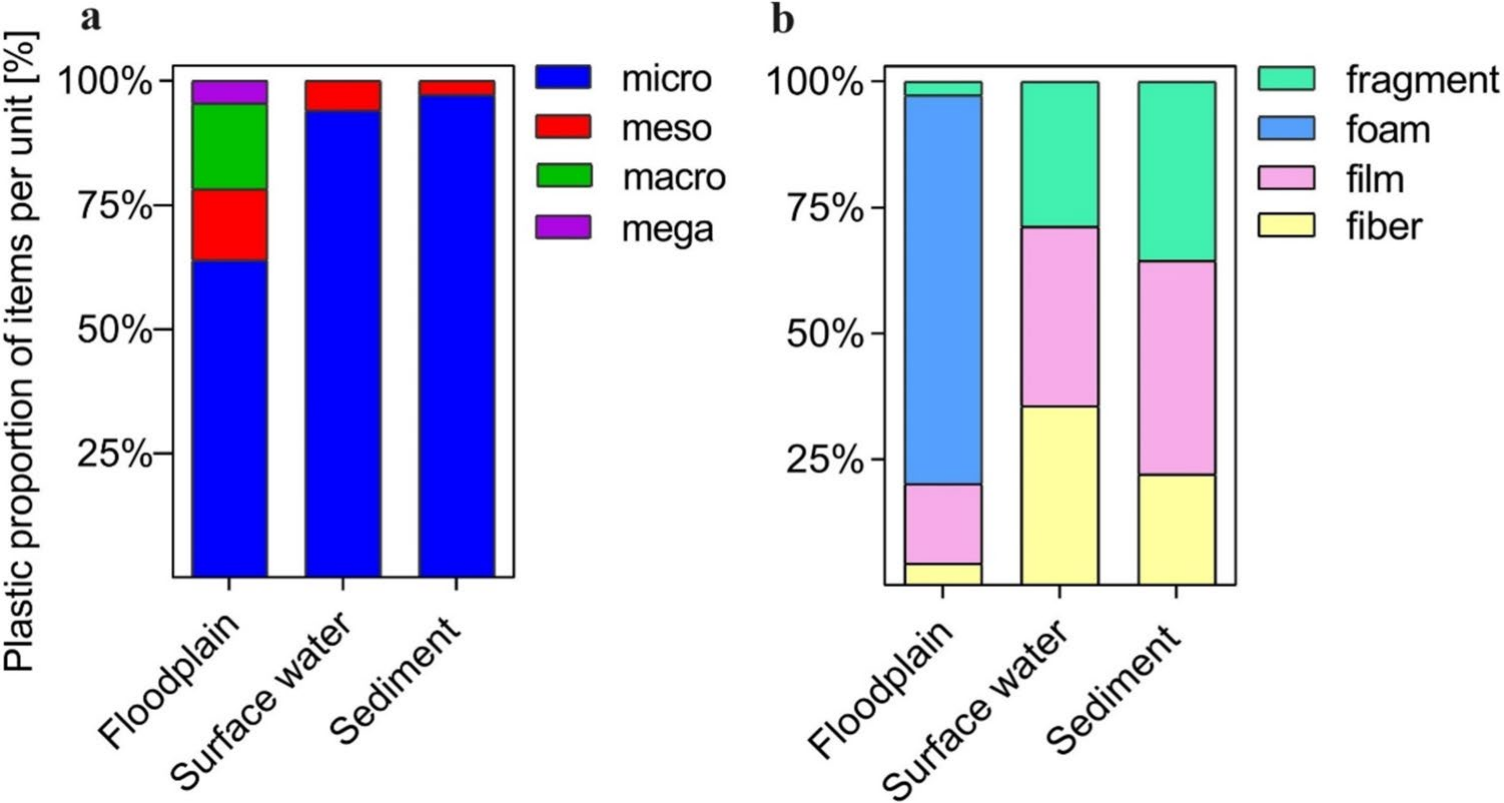
The size and shape distribution of plastic debris in river compartments. Units are expressed as area basis in the floodplain, volume basis in surface water, and weight basis in sediments

Microplastics were dominantly distributed in the surveyed floodplain, surface waters, and sediments (Fig. 2a). Mesoplastics were scarce in surface waters and sediments; however, they were commonly found in floodplains. Macro- and megaplastics were found in floodplains but not in surface water and sediments. The plastic size distribution of the Tuul River floodplain showed a predominance of microplastics and a relatively small number of megaplastics in the previous study (Battulga et al. 2019). The difference in the size composition between the floodplain and the other two compartments was attributed to the difference in the physical and chemical properties of their original materials and degradation stages during their transport processes.

The shape composition of the materials was different between the floodplain and the other two surveyed compartments (Fig. 2b). The floodplain areas were dominated by foams (77.2%), followed by films, fibers, and fragments. The latter three types are evenly distributed in surface water and sediments (Fig. 2b).

**Fig. 3:**
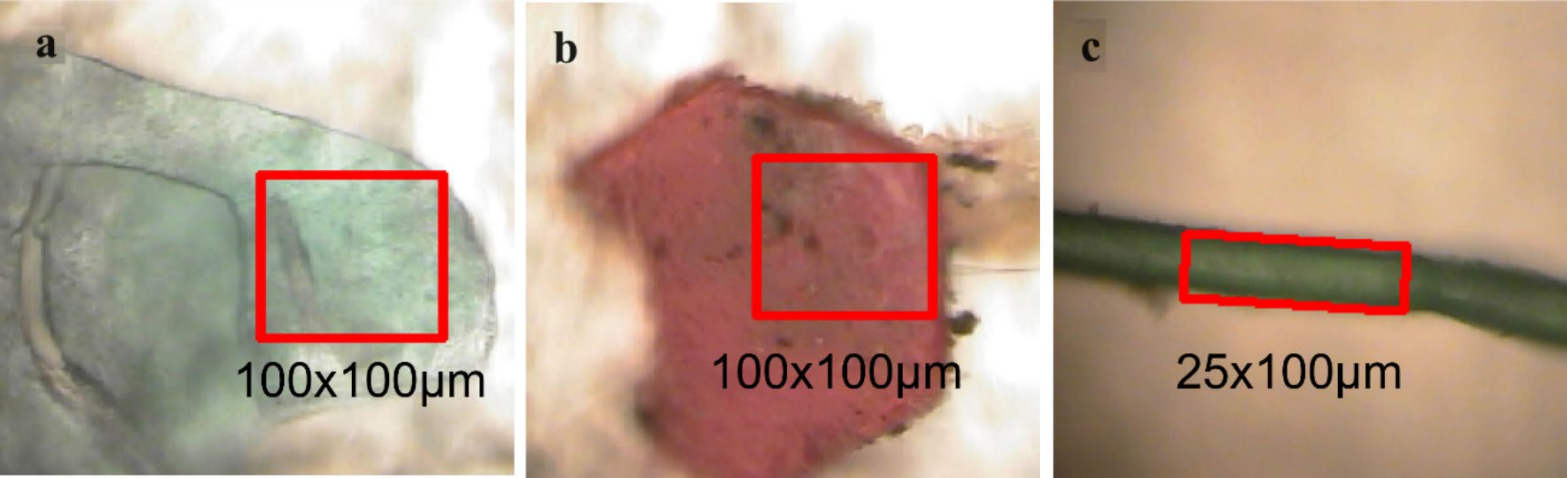
Photoimages of identified microplastic samples from sediments observed using micro-FTIR. **a** Film. **b** Fragment. **c** Fiber. Red squares indicate the focuses of irradiation areas by infrared

**Fig. 4:**
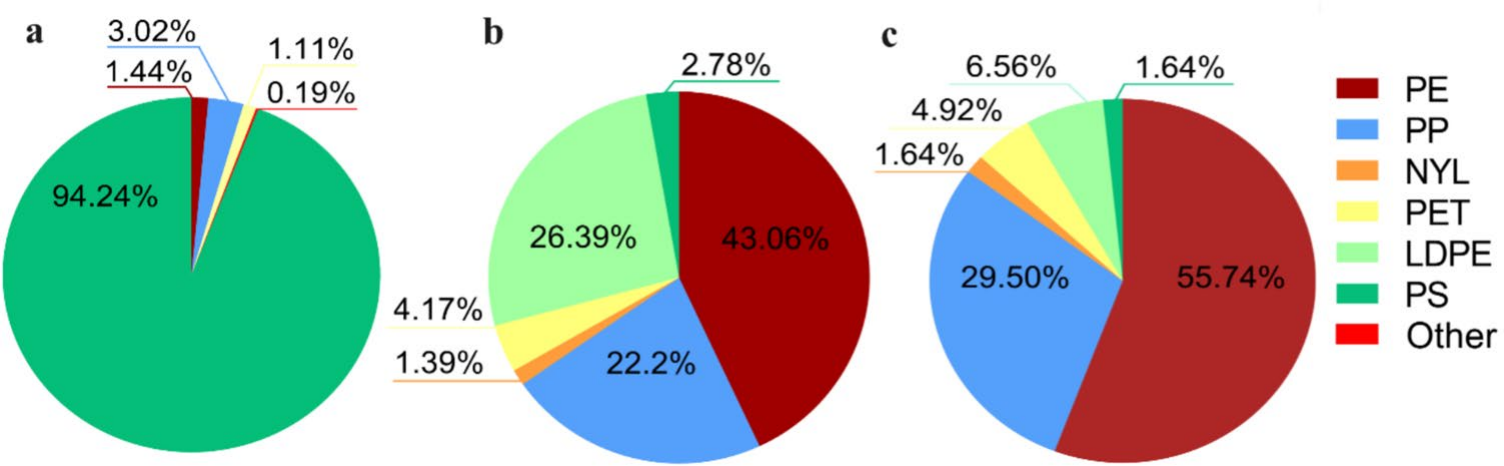
Changes in plastic polymer composition in the river. **a** Floodplain (NYL and PVC were listed as Other). **b** Surface water. **c** Sediment

Seven common types of plastics, namely polyethylene (PE), polypropylene (PP), low-density polyethylene (LDPE), polystyrene, nylon (NYL), polyethylene terephthalate, and polyvinylchloride (PVC), were identified from the total collected samples, which were identified by the micro-FTIR analysis (Figs. 3 and 4). The composition was largely different between the collected plastic debris on the floodplain, in the surface water, and in sediments. The predominance of PSF was characteristic of the floodplain samples, while it was only 2.7% in the surface water and 1.6% in the sediments. PSF has a high buoyancy due to its structure, resulting in the release of PSF into surface water washed ashore along the floodplain, as observed during the field survey (Fig. S1). Additionally, the large consumption of PSF results in the generation of plastic debris in a wide size range as it undergoes degradation. In surface water samples, most of the polymer debris consisted of LDPE, PE, and PP, whereas sediment plastics were dominated by PE and PP. Moreover, the polymer composition of surface water and sediment plastics was correlated significantly (*p* < 0.01). Two major plastics, PE and PP, are common plastic debris in surface water and sediments, indicating that sedimentary plastic debris undergoes density transformation during in-stream processes. PVC was not detected in the surface water and sediment samples analyzed.

### Structural changes on the surface of microplastics

**Fig. 5:**
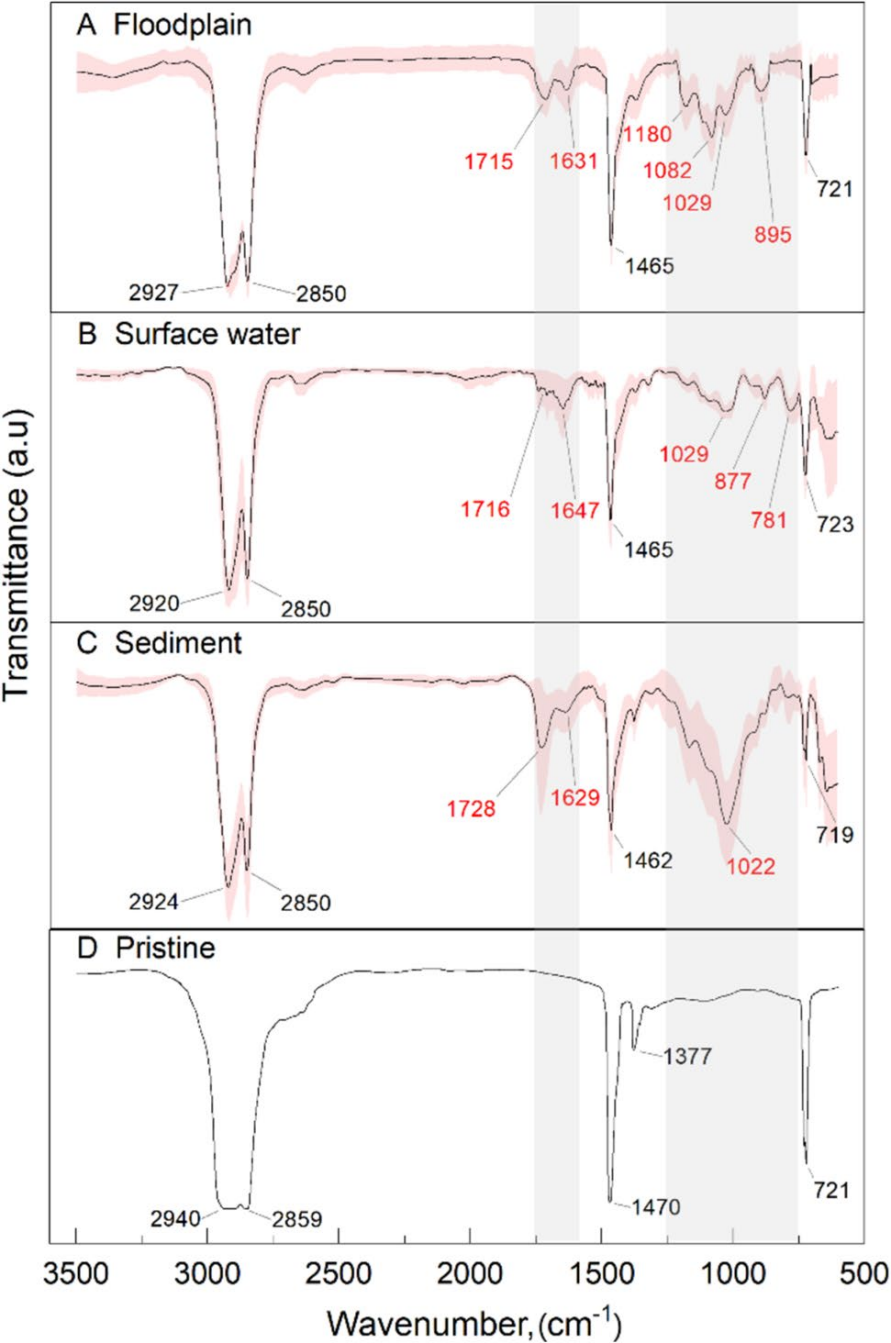
Averaged spectral data of polyethylene (PE) plastics in river compartments. **a** Floodplain. **b** Surface water. **c** Sediment. **d** Pristine PE. Red-marked wavenumbers displayed peaks that were not present in the pristine PE indicated in the grey zone

PE was the most representative and comparable plastic type in river compartments, and the CI was calculated using total of 75 PE samples from each compartment (25 pieces). The average FTIR spectra of PE are shown in Fig. 5. The spectra of pristine PE were obtained from the Shimadzu library (Fig. 5d).

In comparison to the pristine spectra, degraded plastics in the field exhibited multiple peaks, as shown in Fig. 5a, b, and c. In particular, the C = O double bond peak appeared on most of the samples observed at 1715–1730 cm^−1^, which indicated the photodegradation process of saturated aliphatic moieties (Guo et al. 2023). Moreover, a weak spectral band appeared at 1629–1647 cm^−1^ for all three compartment samples, which corresponds to C = O stretching vibrations or the amide I band (Celina et al. 2021).

Floodplain samples exhibited C–O stretching vibrations, which appeared at 1180, 1082, and 1029 cm^−1^, and C–H bending vibrations at 895 cm^−1^ corresponding to the C–H stretching vibrations at 2920 and 2850 cm^−1^ (Tofa et al. 2019).

In surface water samples, C–O stretching vibrations appeared at 1029 cm^−1^, and bands appeared at 877 cm^−1^ and 781 cm^−1^, which might be attributed to unsaturated groups during photodegradation, respectively (Gardette et al. 2013; Yagoubi et al. 2015; Tofa et al. 2019).

Sediment plastics showed a strong absorption at 1022 cm^−1^, which can be attributed to silica oxide stretching vibrations (Hahn et al. 2019). This indicates that sedimentary minerals may reside on the surface of the plastic debris and form plastic-associated minerals (PAM) during the accumulation process.

### Photodegradation stages

**Fig. 6:**
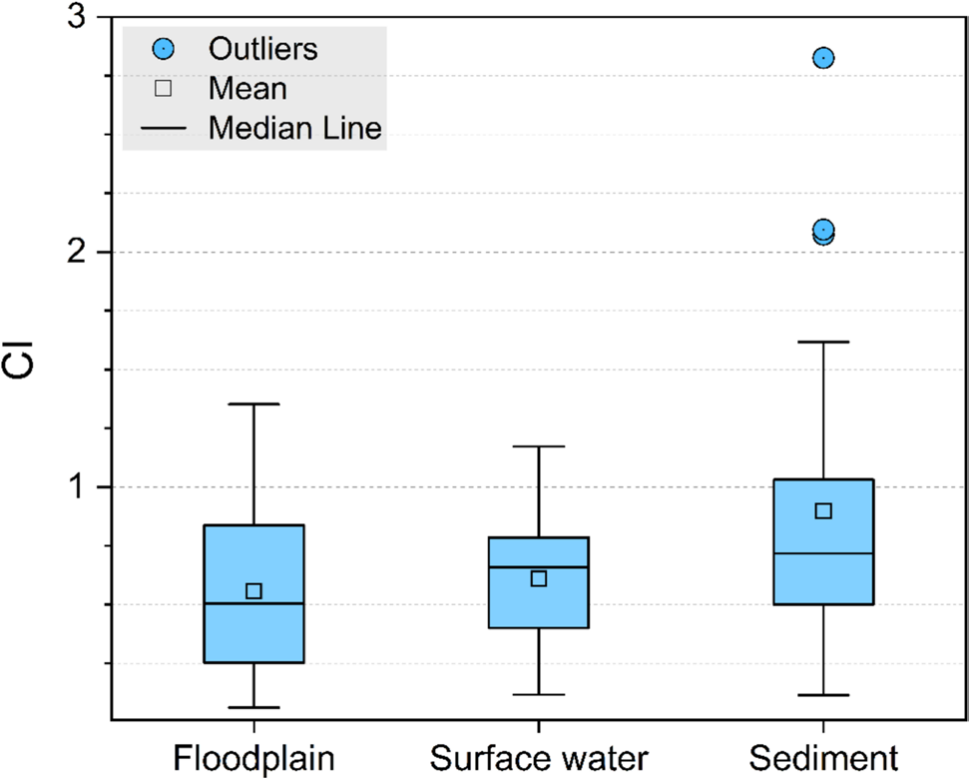
Carbonyl indices of the PE microplastics. The upper and lower whiskers of the boxplots present the 10th to 90th percentiles, and the box shows the 25th to 75th percentiles of the data

The CI value of the floodplain PE plastics was 0.56 ± 0.35. A slightly higher CI value (0.61 ± 0.26) was found in the PE in the surface water. The highest average value of CI (0.90 ± 0.68) was recorded in the PE in the sediments (Fig. 6). There was a statistically significant (*p* < 0.05) difference between the PE in the three river compartments, as observed by one-way ANOVA. The highest CI value reached 2.83, recorded in the sedimentary PE, whereas the lowest CI was 0.06 in the PE in the floodplain. Although there was a slight difference in the average CI values of the PE in the floodplain and surface water, the sedimentary PE indicated a highly deteriorated surface with high CI values.

The difference in the CI between the river compartments was not well recognized in other studies. A few studies report the CI values for naturally weathered plastics in river water and sediment. Rodrigues et al. (2018) reported two times higher CI average values for the PE in sediments than for those in surface water, indicating that relatively deteriorated PE could reside in sediments.

Among the four shapes, except for foams, the CI of fragment plastics was the highest (0.75 ± 0.06), followed by fiber (0.67 ± 0.15), and that of film was the lowest (0.62 ± 0.24). Fragments are typically bulk and solid plastics compared with fibers and films, which have thin and narrow shapes. Fragment plastic can be more durable than that of film and fibers. Therefore, its high CI values may be related to its long durability in the environment.

## Discussion

### The differences of plastic composition in river compartments

On the floodplain, PSF was the major plastic debris because PSF boards are used for heat insulation materials as Mongolia has a harsh continental climate. Similarly, construction PSF dominance as major plastic debris was recorded in the cold region, Canada (Gao et al. 2023). The dominant polymer type of the area may be related to their specific use in the research region. The composition of plastic debris in the floodplain differed from that of the other river compartments in two specific ways. First, the composition in size, shape, and polymer types of plastics in the floodplain was more diverse than those in the other two river compartments. This diversity can be attributed to the different sources and transport pathways of plastic debris to the floodplain, such as wind, high precipitation, and littering of the river. The relatively large plastic debris degrades by mechanical degradation by wind (Chubarenko et al. 2016) and freeze–thaw cycles (Koutnik et al. 2022) during their transport to the floodplain, accompanied by a size reduction process. The size reduction process initiated in the floodplain increases the plastic surface area and surface roughness (Carson et al. 2013; Nava and Leoni 2021), which leads to the formation of an active surface on the plastic debris. Second, the repeated movement of plastic debris between the surface water and the floodplain zone is caused by water level changes or strong water turbulence. The repeated inundation and wash-up of plastic debris in the river ashore will promote changes in the surface properties and degradation of plastic debris.

The similar composition of plastic size, shape, and polymer types in surface water and sediment compartments indicated the interrelationship between those compartments. Especially, most of the plastics found in the surface water were also found in the bottom sediment, allowing us to assume on the deposition process of plastic debris from the river surface to the bottom sediment despite their potentially lower density than water. This hypothesis is supported by the significant correlations (*p* < 0.01) of polymer composition between water and sediment compartments. Consistent with our findings, PE and PP plastics were the most dominant plastic types in river water and sediment worldwide (Tables 1 and 2) (Curren et al. 2021; Bai et al. 2022). The abundant distribution of these plastic types is probably related to their common uses and less cautious management.

### Plastic interactions and aging in river compartments

The original density of PE ranges between 0.88 and 0.92 and that of PP ranges from 0.83 to 0.92 g/cm^3^ (Andrady 2017; Curren et al. 2021). Although low-density plastic is a major component in the bottom sediment of global rivers (Razeghi et al. 2021), the accumulation process in river bottom has not been explained yet. Some studies have briefly mentioned that plastic density can change due to the formation of aggregations and microbial associations (Frei et al. 2019; Nguyen et al. 2020).

**Fig. 7:**
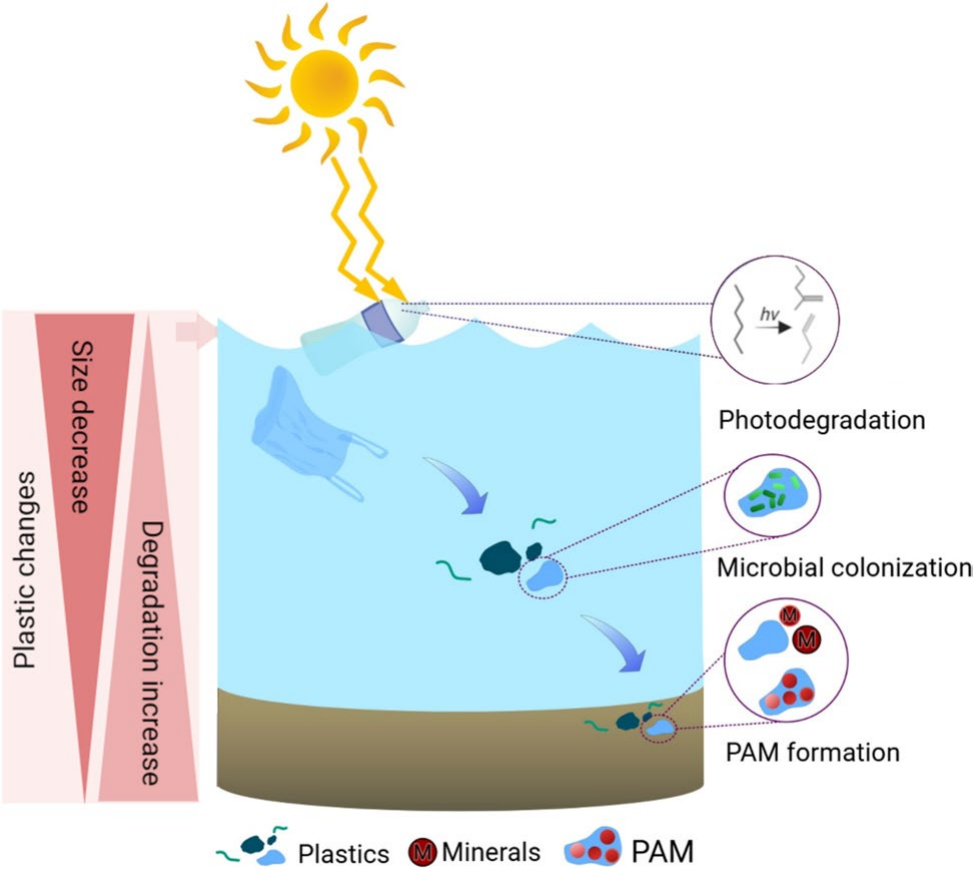
Plastic dynamics in a water column. PAM, plastic-associated minerals

The sinking process of plastic debris in the water column is illustrated in Fig. 7. Light-density microplastics in sediments can change their physical properties, which affect their density during the degradation process. The high CI values of the sediment plastic materials in this study suggest that they have been exposed to ultraviolet radiation for a relatively long period in the environment (Liu et al. 2019; Syranidou et al. 2023). Therefore, the sedimentary PE plastics are more degraded or “older” than the same plastic debris in other compartments. The aging process of plastic may start from floodplain compartment by interacting with surrounding factors including solar radiation, freeze–thaw cycles, and mechanical degradation. Subsequently, in the water compartment, continuous ultraviolet radiation leads to surface deterioration on the plastic, resulting in biotic and abiotic interactions with chemicals and microorganisms, leading to microbial colonization (biofilm-coating) and biofouling (Nguyen et al. 2020). The degraded surface of plastic debris is a favorable condition for biotic and abiotic interaction due to the increased surface area and the change in chemical properties from a hydrophobic to a hydrophilic surface, which transforms non-reactive chemical moieties into reactive functional groups (Ren et al. 2021; Andrady and Koongolla 2022). When the plastic debris reaches the sediment zone, the biofilm-coated plastics participate in a plastic–microorganism–mineral cycle association, as there are three phases of interaction between plastic, sedimentary microorganisms, and minerals. The surface-changed plastics may enable the capture of minerals in river bottom sediments. Apart from plastic debris sedimentation, mineral and microbial interactions occur naturally in the sediment (Dong et al. 2022). The strong absorption intensity of silicate minerals was detected in sediment PE (Fig. 5c). However, the absorption peak was not observed in the PE in floodplain and surface water compartments.

Finally, the consequences of photodegradation, mechanical degradation, and microbial colonization accompanied by mineral association altered the plastic density, leading to a sinking process over time. Therefore, plastic debris in the river sediment might have a high CI because of aging. Low-density plastics, such as PE, LDPE, and PP, sink into the river bottom zone by increasing their plastic density through biotic and abiotic interactions in the stream process. A laboratory-conducted study supported the density increase of PP by microalgae colonization (Lagarde et al. 2016). Similarly, Waldschläger et al. (2020) mentioned that biofouling and environmental weathering alter plastic density, influencing vertical transport within the water column (Waldschläger et al. 2020). However, due to the complex structure of plastic debris, it is technically difficult to determine the apparent density of microplastic. An exposed surface of the plastic debris is required to identify the origin of plastics by spectral analysis. The removal of the surface cover is a common process for the chemical identification of microplastics, which directly affects the determination of the apparent density of plastic debris after the removal of the biofilm coating.

The density-changing process that occurs on plastic debris can be a general phenomenon in global river systems, as the dominant polymer occurrence is the same as in this study (Kumar et al. 2021; Curren et al. 2021; Bai et al. 2022). Despite the similarity in plastic composition between river water and sediments, significant differences in plastic degradation states (CI values) are observed from the surface to the bottom.

## Conclusion

This study was the first attempt to present plastic photodegradation in three compartments of the river system in different size ranges from micro to mega. Microplastics were the most abundant in the size category across all compartments. Notably, floodplain areas have a considerably heterogeneous distribution in terms of size, shape, and polymer types. The similar occurrence of plastic composition suggests a dynamic interrelationship between surface waters and bottom sediments, as indicated by significant correlations in polymer composition in microplastics. Despite the similarities, the degradation state was significantly higher in the sediment PE, indicating that it had been exposed to UV radiation for a long period of time prior to accumulation in the sediment. This prolonged exposure led to the density-changing process, resulting in the accumulation of low-density plastics in the sediment compartments. The density-changing process can affect the dynamics and lifetime of plastic debris in the system.

## Supplementary information

Below is the link to the electronic supplementary material.

Supplementary file1 (DOCX 480 KB)

## Data Availability

The datasets of this study are available from the corresponding author upon reasonable request.
