# Supplementary figures and images for: Dynamics of plastic debris and its density change between river compartments in the Tuul River system, Mongolia
Source: Environ Sci Pollut Res Int. 2024 Nov 26;31(57):65548–58. doi: 10.1007/s11356-024-35584-w (PMC11632066; doi:10.1007/s11356-024-35584-w)

**Supplementary material**


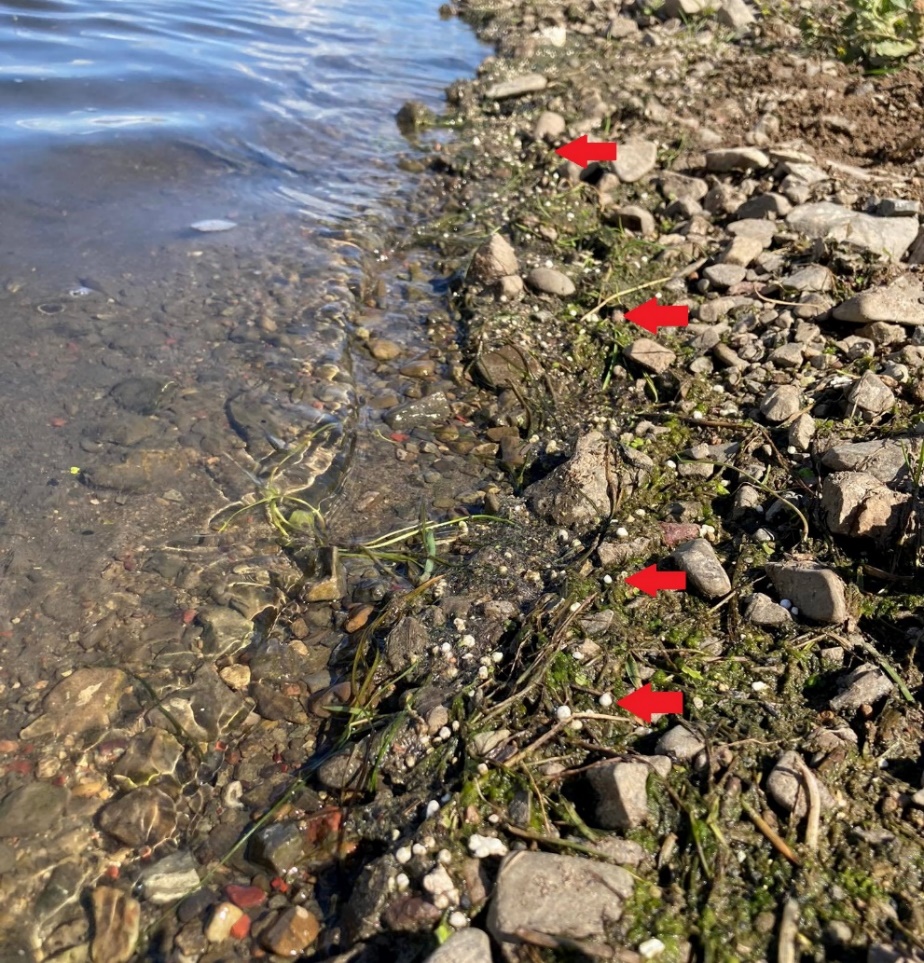


Fig. S1. Washed-up PSF on river edge (indicated by red arrows)

Supplement: Supplementary file 1 — Supplementary file1 (DOCX 480 KB) [file 11356_2024_35584_MOESM1_ESM.docx]
